# The Institute for Medical Technology Assessment Productivity Cost Questionnaire (iPCQ) and the Medical Consumption Questionnaire (iMCQ): Translation and Cognitive Debriefing of the Arabic Version

**DOI:** 10.3390/ijerph18147232

**Published:** 2021-07-06

**Authors:** Sinaa A. Al-Aqeel, Shiekha S. AlAujan, Saja H. Almazrou

**Affiliations:** Clinical Pharmacy Department, College of Pharmacy, King Saud University, Riyadh 14511, Saudi Arabia; salaujan@ksu.edu.sa (S.S.A.); salmazrou@ksu.edu.sa (S.H.A.)

**Keywords:** costs and cost analysis, surveys and questionnaires, translating

## Abstract

The aim of this study was to translate the Institute for Medical Technology Assessment Productivity Cost Questionnaire (iPCQ) and the Medical Consumption Questionnaire (iMCQ) from English into Arabic and perform cognitive debriefing in a Saudi Arabian setting. We conducted the translation according to guidelines, including two independent forward translations and a backward translation. Cognitive debriefing was carried out in two stages. First, the pre-final translated versions of the two questionnaires were tested on a group of respondents (*n* = 5) using face-to-face or telephone interviews. The participants completed a copy of the questionnaires, identified items or questions that were confusing or misunderstood, and then answered a series of open-ended questions about their understanding of each instruction, question and response option. Second, another group of participants (*n* = 17) completed the questionnaire and circled any word that was confusing or difficult to understand and provided comments on the questionnaires. The Arabic translation and linguistic validation were realized without any major difficulties. The few changes made after cognitive debriefing generally related to changing one word to a more appropriate Arabic word. The final Arabic translation needs to be validated for psychometric properties such as validity and reliability before being recommended for use in future research.

## 1. Introduction

The measurement of costs by determining the quantity of resources utilized and then the valuation of costs by assigning a unit cost or price to resource items are central steps in any economic evaluation. Information on the type and quantity of resources utilized could be obtained from medical records, administrative data, disease registries, self-reported data and expert opinions. Each of these methods has advantages and disadvantages [1,2,3]. Questionnaires, interviews, and diaries are examples of self-reported methods for measuring resource use data. Patient-completed questionnaires are a reliable method of measuring estimates of resource utilization [1,2,4,5,6]. 

Despite the growing interest in the role of economic evaluation in the decision-making process in Arab countries, the published literature is limited in quality and quantity [7,8]. The availability of cost and effectiveness data has been reported as the main barrier to conducting economic evaluation by researchers in the region [9]. 

We postulate that the availability of valid and reliable Arabic language questionnaires to measure resource utilization would allow for a generation of economic evidence. This involves either developing or translating appropriate questionnaires. Accurate translation and appropriate cultural adaptation of a questionnaire allow a comparison of findings between populations speaking different languages. Hence, the aim of this study was to translate the Institute for Medical Technology Assessment [10] Productivity Cost Questionnaire (iPCQ) and the Medical Consumption Questionnaire (iMCQ) from English into Arabic. The translation of these questionnaires was in preparation for a larger study that will be conducted to measure resource use among Saudi Arabian populations. 

The iPCQ measures and values health-related productivity loss due to illness, disability or psychiatric problems, while the iMCQ measures patients’ healthcare consumption [10]. There is no consensus on the best instrument for measuring productivity loss [11]. The iCPQ has several advantages. The iPCQ is suitable for quantifying presenteeism at and absenteeism in paid work as well as productivity losses related to unpaid labor. Furthermore, iPCQ is suited for measuring and valuing productivity losses for use in economic evaluations of healthcare [12]. Both the iPCQ and iMCQ are non-disease-specific instruments suitable for online use and face-to-face interviewing [12]. The iPCQ and iMCQ consist of 18 and 20 questions, respectively. The two questionnaires are available in different languages including Dutch, English, French, German, Norwegian [13] and Korean [14]. 

## 2. Materials and Methods

We acquired the approval from the Institute for Medical Technology Assessment for the translation of the Arabic version of the iPCQ and iMCQ. 

The methodology to conduct the translation and linguistic validation was based on the International Society for Pharmacoeconomics and Outcomes Research [ISPOR) principles of good practice for the translation and cultural adaptation process for patient-reported outcome measures [15] and the guidelines for the process of cross-cultural adaptation of self-report measures [16]. The study proposal was reviewed by the Institute of Review Board, King Fahd Medical City (IRB log number 20-532E) and deemed to be exempt from ethical review. Informed consent was obtained from all subjects involved in the study. 

### 2.1. Forward Translation

The English language source versions were forward-translated to Standard Arabic independently by two translators; one is a local professional translator with no medical or clinical background, and the other has a pharmacy background with doctoral training in health economics (author, S.A.A.-A.). Both translators are native Arabic language speakers and are fluent in the English language. 

The expert committee consisted of two of the authors (S.H.A., S.S.A.), who have a background in pharmacy and doctoral training in health economics, and the translators reviewed and discussed deviations between the two versions until consensus on one reconciled version for iPCQ and iMCQ was reached.

### 2.2. Backward Translation 

One professional translator blind to the original English version of the questionnaires performed a backward translation of the reconciled version of the two questionnaires back into the source English language. The back-translated versions were compared with the original English questionnaires, and discrepancies were noted by one author (S.A.A.-A.) and reviewed with the committee to ensure conceptual equivalence between the translation and source text. This step resulted in a pre-final version for iPCQ and iMCQ. 

### 2.3. Cognitive Debriefing 

Cognitive debriefing to assess the level of comprehensibility and cognitive equivalence of the translation was carried out in two stages. First, the pre-final translated version of the two questionnaires was tested on a group of participants (*n* = 5) using face-to-face or telephone interviews according to the participants’ preference. All participants were asked to complete a copy of the questionnaire. Then, we asked a series of open-ended questions about their understanding of each instruction, question and response option. They were also asked to restate in their own words what they think each translated item meant. A member of the expert committee (S.A.A.-A.) reviewed the results from the cognitive debriefing and identified modifications necessary to improve the performance of the translation in consultation with the committee. Data collection was an iterative process whereby comments from one participant were used to modify the survey for the following respondents. 

Second, a group of participants (*n* = 17) completed the questionnaire and circled any word that was confusing or difficult to understand and provided comments on the questionnaires. After the tenth participant, there were no new issues or difficulties identified (point of saturation); however, seven additional individuals were recruited to confirm the stability of the findings. This step resulted in the final version for the iPCQ and iMCQ. 

The study sample was recruited purposively using a variety of methods including social media (Twitter^®^ and WhatsApp^®^) and the snowballing method. An invitation to join the study was sent via WhatsApp or Twitter. Individuals who were interested in the study were asked to contact the researchers by e-mail or phone. The first group of individuals surveyed were asked to recommend others to participate. The inclusion criteria were being a member of the target population (Saudi Arabian) and able to read in Arabic. 

### 2.4. Proofreading 

An Arabic language expert reviewed the final Arabic translation for errors of spelling, grammar, punctuation and typography. The final Arabic translation was compared with the English version by a professional translator to ensure conceptual equivalence. The final version of the iMTA and iPCQ are available upon request from the Medical Technology Assessment. 

## 3. Results

### 3.1. Forward Translation

The education levels were modified to account for the Saudi education system using the standard levels used in the General Authority for Statistic surveys. The word ‘partner’ in the iPCQ question number 12 was translated into wife as it culturally not acceptable to have a partner without marriage in Saudi Arabia. 

In the instructions section of the English versions, the words ‘the questionnaire’, ‘the form’ or ‘the list’ were used to describe the questionnaire and were translated into one word (aistibyan), meaning questionnaire, to maintain consistency in describing the instrument. 

The words’ translation was almost identical between the two translators but the sentence structure differed in some instances. In this case, the committee selected the most grammatically accurate sentence structure. For the iPCQ, the differences in word selection were the word ‘bothered’ in question 7, 8 and 9. One translation was ‘suffered’ (aaniat) the other was ‘annoyed’ (Aizeajatak). There was disagreement between the committee members on which translation was most conceptually similar to bother. The decision was to use the professional translator’s version (‘suffered’, aaniat) and revise the translation after the backward translation. In the same questions, the word ‘problems’ was translated into the word ‘ailment’ (aietilalat) by one translator and ‘problems’ (mashakil) by the other; the second translation was used. For the iMCQ, one difference in word selection was the translation of the instruction ‘Otherwise skip’; one translator translated it into ‘if No, skip’ the other translation was ‘Otherwise skip’, and the latter was selected by the committee. The other difference was the word ‘daycare’ in questions 15a and 16a. One translation was ‘rieayat niharia’, the other ‘alyawm alwahid’. As both translations are accepted, the committee’s decision was to use the professional translator’s version ‘rieayat niharia’ and revise the translation after the backward translation and cognitive debriefing. The other difference was in the translation of the word ‘rehabilitation’; one translation was ‘habilitation’ and the other was ‘rehabilitation’, and the latter one was considered more accurate and was thus selected. The final difference was in the translation of ‘residential care or nursing home’. One translated residential care literally into ‘rieyea sakania’ and nursing home into ‘care home’ (dar rieaya); neither reflect the conceptual meaning of the English version. The second translation was ‘long-term care facility’ to describe all services that assist with medical and non-medical needs, including residential care and nursing homes. The committee agreed that the second translation was more appropriate for our setting and reflected the purpose of question 16, which asks about institutions other than the hospital for daycare treatment. 

The committee reached a consensus on one reconciled forward translation (version 1) for the iPCQ and iMCQ, ready for back translation. 

### 3.2. Backward Translation

The majority of discrepancies between the original English and the back-translated versions were related to the use of different words that convey the same meaning. Few discrepancies were related to differences in verb tense, for instance, ‘did you use’ became ‘have you used’. In the iPCQ questionnaire, one word from the back translation with a different meaning than the English version was retranslated. This word was ‘bothered’ in questions 7, 8 and 9. After discussion, the committee agreed that retranslating the word into ‘annoyed’ (Aizeajatak) was more appropriate. In the iMCQ questionnaires, the translation of ‘Cesar therapist, Mensendieck therapist or a manual therapist’ was different from the original English version but conveyed the same meaning as someone specialized in posture and movement. The backward translation was useful in detecting if an extra word was added; for instance, in question 9 in the iPCQ, the word ‘work’ was back translated into ‘work task’, and the Arabic translation was revised to delete the word ‘task’. The translation revision resulted in a pre-final version (version 2) for the iPCQ and iMCQ, ready for the cognitive debriefing. 

### 3.3. Cognitive Debriefing

Two participants raised a comment about the word school in ‘I am a student, I go to school’, one of the answer options for question A5 about ‘what do you do’. In our setting, the word school is associated with pre-university education; therefore, university students may find this option confusing. We deleted ‘I go to school’, and the sentence became ‘I am a student’. In both questionnaires, some respondents gave suggestions for the format of the questionnaire, such as using underline or using a table instead of a list. We documented these recommendations, but no format changes were made. The following sections and Table 1 and Table 2 outline major issues identified by respondents within the two questionnaires.

#### 3.3.1. iMCQ

In the English version iMCQ, the word ‘care’ was used without specification in two occurrences: ‘Questionnaire about your use of care’ and in the instruction section ‘The questionnaire is about your use of care’; some respondents found this confusing. Elsewhere in the English version the word healthcare is used; therefore, we changed the translation of ‘care’ into ‘healthcare’ in these two occurrences to eliminate any confusion. The translation of ‘consultations count,’ ‘consultations do not count’, and ‘daycare’ were revised based on the participants’ comments. 

Few respondents expressed difficulties in understanding the meaning of homeopath, Caesar therapist, Mensendieck therapist and manual therapist. We acknowledge the relative unfamiliarity of these occupations in our setting; however, the committee agreed that this is the best translation available, and for this reason did not change the translation. Some respondents did not understand the meaning of occupational therapist or asked if a psychotherapist is the same as a psychologist. The committee agreed that the confusion was not caused by the translation but by the unfamiliarity with these terms; as all translations of occupations were specifically checked against the official translations used in academia and official job titles in relevant organizations. We expected those who received occupational therapy or psychotherapy to be familiar with these terminologies; therefore, no changes were made.

Several interesting comments were made by the participants, not related to the translation, but highlighting useful points to be considered by researchers interested in using the questionnaire. These included the language in which the medications’ names should be written (English or Arabic) and if the word ‘treatment’ in iMCQ question 15b refers only to medication or any type of treatment. 

A comment was made about the sentence ‘a period of 3 months counts as 13 weeks’. In Saudi Arabia, the use of the Hijri (Lunar) calendar of 12 months each with 29 or 30 days is common, which might cause this confusion as three months counts as 12 weeks. However, as the payroll is based on the Gregorian calendar, the committee agreed no change was required.

#### 3.3.2. iPCQ

Three respondents commented that the meaning of the word productivity was not very clear to them. As the confusion was not caused by the translation of ‘productivity’ but by the participants’ unfamiliarity with the word in Arabic, no changes were made.

Two respondents commented on the determination of the disability percentage in the answer option ‘I am unable to work, for …. %’ in question A5. The Saudi General Organization for Social Insurance defines total occupational disability as a disability that totally and permanently prevents the contributor from engaging in any work or profession with a disability percentage of 100%; a partial disability degree is ≥50% but less than 100%, and reduces the earning capacity of the contributor. Such percentages are determined by the appropriate Medical Board. The committee agreed no change was required as participants officially considered as disabled are familiar with this terminology and will find no difficulty in answering this question. 

There was a suggestion to replace the word ‘occupation’ with the word ‘job’; we revised the translation and used an alternative for the word ‘occupation’, but not the word ‘job’.

Some comments suggested that question 9 was composed of two separate issues: ‘was it perhaps difficult to get as much work finished as you normally do? On these days how much work could you do on average?’, while the answer option available relates to the second issue. We changed ‘was it perhaps difficult’ to’ perhaps it was difficult’ and replaced the first question mark with a comma to better fit Arabic grammar. 

## 4. Discussion

This translation and cognitive debriefing study represents the first translation of the iMTA and iPCQ into the Arabic language. To our knowledge, this also the first translation of any productivity loss or resource use instrument into the Arabic language. The results of the cognitive debriefing interviews indicated that the translations of the two questionnaires into the Arabic language adequately captured the concepts of the English language version of the questionnaires and were readily understood by the participants. The translation of the iPCQ and iMCQ into Standard Arabic means the questionnaires could be used as reference material for subsequent adaptations in other countries where the Arabic language is used. The availability of Arabic translations of the iPCQ and iMCQ is expected to facilitate costing studies in the region and to stimulate health economics research. 

A major strength of this study was following established guidelines in the translation and linguistic validation process. The sample size (*n* = 22) used for the cognitive debriefing was comparable to that in published research of a similar nature [17,18,19,20], although lower than the recommended sample size of 30–40 [14,15]. The study’s major limitation is the recruitment of a highly educated sample all with a university degree. A more robust approach would be to interview people with different education levels; however, using the convenience sampling method and social media advertising skewed our sample towards the highly educated. Fortunately, the original questionnaires were written in a language level that text can be comprehended by 95% of the general population, including those with lower education levels [12], which could mitigate this limitation in our study. 

According to guidelines, the back translations should be produced by two persons with the source language (English) as their mother tongue [14]. In our study, the back-translator’s native language was Arabic because of the scarcity of native English language official translators in our setting. The use of a non-native back-translator might introduce an information bias and result in unexpected meanings being neglected [21]. We only performed one backward translation to utilize resources that would be allocated to a second translator elsewhere in the development of the translated version. Some guidelines suggest that a single back translation is adequate when supplemented with an expert panel [15,21] which was the case in our study. 

One of the translations that was revised as a result of the back translation was the word ‘bother’ in the iPCQ. Achieving the conceptual equivalence of the English word ‘bother’ is difficult because of the wide range of definitions and interpretations of ‘bother’ across languages and cultures [22]. The cognitive debriefing stage revealed no difficulty in understanding the meaning of the word bother. 

The original questionnaires were directed to the Dutch healthcare setting; therefore, some resources examined may not be widely used in other countries. In our study, this was observed with resources such as homeopaths, Caesar therapists, Mensendieck therapists and manual therapists. The unfamiliarity with these therapists created some confusion among the participants, but we expected that those who had received such therapy would be familiar with the terminology; therefore, no changes were made to the translated questionnaire. 

We recommend that researchers interested in using the questionnaire in our setting provide a simple explanation in a separate letter accompanying the questionnaire. This includes, for example, the definition of certain terms such as productivity and preferences for writing the name of medications in English or Arabic. 

In this study, we focused on the translation process for the iPCQ and iMCQ. The final Arabic translation needs to be validated for psychometric properties such as validity and reliability before being recommended for use in future research. The COnsensus-based Standards for the selection of health status Measurement Instruments [COSMIN] checklist can be used as guidance for designing and reporting such studies on measurement properties [23,24]. 

## 5. Conclusions

We produced an Arabic translation of the iMTA and iPCQ questionnaire. The final Arabic translation needs to be validated for psychometric properties such as validity and reliability before being recommended for use in future research.

## Figures and Tables

**Table 1:** Issues and changes in the iMCQ.

Category	Comment (Number of Respondents)	Changes
Instructions/ explanations	What care? (*n* = 5)	Revised to ‘healthcare’.
	The words ‘consultation count’ and ‘do not count’ are not clear (*n* = 2)	Revised.
	Why are explanations in a question format (interrogative) rather than a statement sentence? (*n* = 1)	No change.
Questions		
QA5	‘I go to school, I am a student’ What about university students? (*n* = 2)	Deleted ‘I go to school’, and the sentence became ‘I am a student’.
Q3	What do you mean by Cesar therapist (*n* = 2), Mensendieck therapist (*n* = 1) or a manual therapist? (*n* = 1)	No change.
Q4	What do you mean by occupational therapist? (*n* = 3)	No change.
Q7	Homeopath (*n* = 15)	No change.
Q8	Is psychotherapist the same as psychologist? (*n* = 1)Replace psychologist or a psychotherapist or psychiatrist with providers of psychiatric care (*n* = 1)	These are different specialists and cannot be combined. No change.
	Suggested re-writing ‘… psychologist or a psychotherapist or psychiatrist in the past 3 months?’, instead of ’… psychologist in the past 3 months? Or with a psychotherapist or psychiatrist?’ (*n* = 1)	No change.
Q9	I do not have a (company doctor), how can I answer this question? (*n* = 2)	No change.
10c and 19c	3 months is 12 weeks not 13 weeks (*n* = 1)	No change.
Q11b	‘Medication that you have bought at the pharmacy’ should be ‘that you have bought without a prescription from a pharmacy’ (*n* = 1)	No change was made as people may buy prescription medication from a pharmacy.
Q11b	Should I write the medication’s name in Arabic or English? (*n* = 1)	No change.
Q15 and Q16	Day care (*n* = 4)	Revised.
	Residential/care centre (*n* = 1)	Revised.
Q15 b	Treatment means only medication or any type of treatment? (*n* = 1)	No change.
Q19C	Practical help should be personal help (*n* = 2)	No change as in the same question we have personal care, and changing practical to personal may create confusion.
Q19 b	Change from domestic help to domestic care (*n* = 1)	No change.
Q19C	What if it was for a few days not weeks?	No change.
Q20b	Suggested asking about the time not distance (*n* = 1) Suggested one-track instead of one-way(*n* = 1)	No change as time depends on many factors other than distance.
	The numbering of question 1 is followed by a letter, but the other questions are not?	No change.
General comments	For some answers, use tables or bulletpoints to make it easier to use, e.g., Q10c and Q15b. (*n* = 2)	No change.
	The section ‘What should you do with the questionnaire?’: move to the beginning of the questionnaire so nobody can miss it. (*n* = 1)	No change.

**Table 2:** Issues and changes in the iPCQ.

Category	Comment (Number of Respondents)	Changes
Title	What does productivity mean? (*n* = 3)	No change.
Questions		
A5	I am unable to work, for …. %, how can I determine the percentage? (*n* = 2) What if partially and I can work, can I choose two answers? (*n* = 1)	No change.
Explanation	Do students complete the questionnaire? Unemployed? (*n* = 1)	The instruction is clear ‘if you have a paid job’. Unemployed and students may have financial support from the government but they do not have a paid job. Therefore, no change.
	Explanation box: move to directly under Q 6	No change.
Q1	Should I write my occupation or qualification? (*n* = 1) Use the word ‘job’ instead of ‘occupation’ (*n* = 1)	The word is clear about occupation, not qualification. No change. Revised. An alternative for the word occupation was used but not the word ‘work’.
Q4	The explanation ‘Only count the missed work days in the last 4 weeks’ under ‘Yes’, answer choice is a repetition of the question stem. (*n* = 1)	No change.
Q5	Underline the period before the 4 weeks (*n* = 1)	No change.
Q6	The question is too specific, maybe we cannot remember? (*n* = 1)	No change.
Q9	This is two questions in one? The answer is related to the second question only (*n* = 4)	Revised.
Q12	Give examples of types of help	It is already provided in the explanation box. No change.

## Data Availability

The data presented in this study are available on request from the corresponding author.
